# High prevalence of thalassemia in migrant populations in Guangdong Province, China

**DOI:** 10.1186/1471-2458-14-905

**Published:** 2014-09-02

**Authors:** Bing Li, Xiao-zhuang Zhang, Ai-hua Yin, Qing-guo Zhao, Li Wu, Yuan-zhu Ma, Ming-yong Luo, Shou-yi Yu

**Affiliations:** Department of Epidemiology, School of Public Health and Tropical Medicine, Southern Medical University, 1838 Guangzhou North Road, Guangzhou, Guangdong 510515 China; Department of Healthcare, Guangdong Women and Children Hospital, Guangzhou, Guangdong China; Department of the Office of the Dean, Guangdong Women and Children Hospital, Guangzhou, 521 XingNan Street, Guangzhou, Guangdong 511442 China; Center of Prenatal Diagnosis, Guangdong Women and Children Hospital, Guangzhou, Guangdong China

**Keywords:** Thalassemia, Globin gene mutation, Prevention, Migrant population, Public health service

## Abstract

**Background:**

The objectives of this study were to estimate the prevalence of thalassemia and to analyze the need for public health services for migrant populations in different cities in Guangdong Province, China.

**Methods:**

A cross-sectional survey was conducted in 21 cities of Guangdong Province. Twenty-three types of a- and β-globin gene mutations were detected in a total of 14,230 pregnant women and 14,249 husbands.

**Results:**

There was a 16.45% prevalence of thalassemia among the 28,479 individuals, and the prevalences of α-, β-, and combined α-/β- thalassemia were 12.03%, 3.80%, and 0.63%, respectively. Compared with the native city residents in the province, the migrants from within the province and the immigrants from outside the province had lower prevalences of thalassemia, but the prevalence values were >11%.

**Conclusions:**

The prevalence values for thalassemia gene mutations were high in all three population groups studied in Guangdong Province. The results indicate that all segments of the Guangdong population should be screened for thalassemia.

## Background

Thalassemia is the most commonly inherited, recessive single gene disease. Globally, approximately 7% of pregnant women carry β- or α zero-thalassemia, or other hemoglobin disorders [1, 2], which imposes an enormous burden on families and society [3, 4]. Thalassemia has become a public health problem in Guangdong Province, China. High prevalences of thalassemia are present in Guangdong populations; 17.70% of pregnant women, 15.94% of their husbands, and 16.03% of neonates are thalassemia carriers [5]. To prevent and control thalassemia major, the government of Guangdong has implemented the Thalassemia Prenatal Prevention and Control Program (TPPCP), which has been supported by annual funding of 35 million RMB since 2011. In 2012, we used data obtained from this program and conducted a baseline survey on the epidemiological status of globin gene mutations in Guangdong.

As a developed area in China, Guangdong Province has a large amount of immigration between cities and between provinces, which imposes significant challenges for the public health services. Most public health services are only offered to native city residents who have a registered household as part of the household registry system. Little is known about the thalassemia prevalence in this and in other types of registered household populations in different cities in Guandong Province. We analyzed the α- and β-thalassemia prevalence in these populations.

## Methods

The tested individuals were from one of three populations. The first population consisted of native city residents who were recorded in the local city household register. They had primary social and health insurance coverage and could obtain aid from the public health program. The second population consisted of migrants from within the province, and who were registered as residing in a household in another city in Guangdong Province. These residents had no primary social and health insurance coverage from the cities in which they were currently residing, and had non-transferable health insurance coverage from the cities or counties in which they were registered. They also could not obtain aid from the public health programs of the cities in which they were currently residing. The third population consisted of the immigrants from outside the province. These individuals were not registered as living in a household in Guangdong Province. They had no primary social and health insurance coverage in Guangdong, did not have transferable health coverage in the province in which they were registered, and could not obtain aid from the public health program in Guangdong.

This study used a cross-sectional survey design and was performed using data from the baseline TPPCP survey of Guangdong. The survey was conducted in the 21 cities of Guangdong Province. One district or county was randomly sampled from each city, and a total of 91 hospitals were selected according to the newborn delivery numbers in the sampled districts or counties. After informed consent was obtained, 3 mL peripheral vein blood (collected in an EDTA tube) was sampled from each pregnant woman and her husband during the delivery period. The samples were temporarily placed into a refrigerator at 4°C, and then mailed on ice by express delivery to the genetics and metabolic diseases laboratory of Guangdong Women and Children Hospital for further analysis. They arrived at the laboratory within 3 days, where they were analyzed for the globin gene mutation. A total of 8,479 eligible blood samples were tested, which included 14,230 pregnant women and 14,249 husbands. The results for the number of samples from each city are presented in Table 1.

**Table 1:** **The prevalence (%) of thalassemia carriers among different household register population of cities of Guangdong, China**

Sampled cites of Guangdong	Native city residents in the province			Migrants from within the province			Immigrants from outside the province			Total			Statistic values
	Tested individuals	Thalassemia carriers	Prevalence (%95 CI)	Tested individuals	Thalassemia carriers	Prevalence (%95 CI)	Tested individuals	Thalassemia carriers	Prevalence (%95 CI)	Tested individuals	Thalassemia carriers	Prevalence (%95 CI)	
East Area													
Chaozhou	737	84	11.40	48	4	8.33	202	15	7.43	987	103	10.44	χ^2^ = 2.91,
Raoping			(9.11, 13.68)			(0.55, 16.11)			(3.83, 11.02)			(8.54, 12.33)	P > 0.05
Jieyang	1318	135	10.24	108	12	11.11	54	5	9.26	1480	152	10.27	χ^2^ = 0.14,
Puning			(8.61, 11.87)			(5.21, 17.01)			(1.57, 16.95)			(8.73, 11.81)	P > 0.05
Shantou	620	46	7.42	800	76	9.50	81	9	11.11	1501	131	8.73	χ^2^ = 2.51,
Jinping			(5.37, 9.47)			(7.48, 11.52)			(4.30, 17.92)			(7.31, 10.15)	P > 0.05
Shanwei	1118	123	11.00	60	9	15.00	51	4	7.84	1229	136	11.07	χ^2^ = 1.49,
Haifeng			(9.18, 12.83)			(6.01, 23.99)			(0.50, 15.18)			(9.32, 12.81)	P > 0.05
Total	3793	388	10.23	1016	101	9.94	388	33	8.51	5197	522	10.04	χ^2^ = 1.17,
			(9.27, 11.19)			(8.11, 11.77)			(5.74, 11.27)			(9.23, 10.86)	P > 0.05
Montain Area													
Heyuan	1346	265	19.69	136	29	21.32	91	15	16.48	1573	309	19.64	χ^2^ = 0.82,
District			(17.57, 21.80)			(14.47, 28.17)			(8.90, 24.07)			(17.69, 21.60)	P > 0.05
Meizhou	934	199	21.31	26	5	19.23	42	8	19.05	1002	212	21.16	χ^2^ = 0.18,
Xingning			(18.69, 23.92)			(4.16, 34.30)			(7.23, 30.86)			(18.64, 23.67)	P > 0.05
Qingyuan	1165	237	20.34	88	15	17.05	92	16	17.39	1345	268	19.93	χ^2^ = 0.96,
Yingde			(18.04, 22.64)			(9.23, 24.86)			(9.69, 25.10)			(17.80, 22.05)	P > 0.05
Shaoguan	981	185	18.86	164	20	12.20	139	17	12.23	1284	222	17.29	χ^2^ = 7.15,
District			(16.42, 21.29)			(7.21, 17.18)			(6.81, 17.65)			(15.23, 19.35)	P < 0.05
Yunfu	1048	265	25.29	295	67	22.71	95	11	11.58	1438	343	23.85	χ^2^ = 9.28,
Luoding			(22.67, 27.90)			(17.96, 27.47)			(5.18, 17.98)			(21.66, 26.04)	P < 0.01
Total	5474	1151	21.03	709	136	19.18	459	67	14.60	6642	1354	20.39	χ^2^ = 11.50,
			(19.95, 22.10)			(16.30, 22.07)			(11.38, 17.81)			(19.42, 21.35)	P < 0.05
The Pearl River Delta													
Dongguan	959	130	13.56	412	76	18.45	111	6	5.41	1482	212	14.30	χ^2^ = 13.38,
District			(11.40, 15.71)			(14.72, 22.17)			(1.22, 9.59)			(12.53, 16.08)	P < 0.01
Foshan	847	123	14.52	466	93	19.96	100	11	11.00	1413	227	16.07	χ^2^ = 8.63,
						(16.35, 23.57)			(4.90, 17.10)			(14.16, 17.97)	P < 0.05
Nanhai			(12.16, 16.88)										
Guangzhou	756	93	12.30	375	64	17.07	96	9	9.38	1227	166	13.53	χ^2^ = 6.40,
Panyu			(9.97, 14.63)			(13.28, 20.86)			(3.57, 15.18)			(11.62, 15.43)	P < 0.05
Huizhou	854	170	19.91	502	69	13.75	118	9	7.63	1474	248	16.82	χ^2^ = 16.33,
District			(17.24, 22.57)			(10.75, 16.74)			(2.86, 12.39)			(14.92, 18.72)	P < 0.001
Jiangmen	1282	231	18.02	121	20	16.53	47	5	10.64	1450	256	17.66	χ^2^ = 1.81,
Xinhui			(15.93, 20.11)			(9.94, 23.11)			(1.87, 19.41)			(15.70, 19.61)	P > 0.05
Shenzhen	194	29	14.95	913	149	16.32	174	14	8.05	1281	192	14.99	χ^2^ = 7.85,
Baoan			(9.96, 19.94)			(13.93, 18.70)			(4.02, 12.07)			(13.04, 16.93)	P < 0.05
Zhaoqing	1143	215	18.81	191	46	24.08	85	15	17.65	1419	276	19.45	χ^2^ = 3.09,
Sihui			(16.56, 21.06)			(18.05, 30.12)			(9.58, 25.71)			(17.40, 21.50)	P > 0.05
Zhongshan	705	85	12.06	174	32	18.39	569	65	11.42	1448	182	12.57	χ^2^ = 6.21,
District			(9.67, 14.45)			(12.66, 24.12)			(8.82, 14.02)			(10.87, 14.27)	P < 0.05
Zhuhai	675	115	17.04	382	72	18.85	106	15	14.15	1163	202	17.37	χ^2^ = 1.40,
Doumen			(14.22, 19.86)			(14.95, 22.75)			(7.55, 20.75)			(15.20, 19.54)	P > 0.05
Total	7415	1191	16.06	3536	621	17.56	1406	149	10.60	12357	1961	15.87	χ^2^ = 37.06,
			(15.23, 16.89)			(16.31, 18.81)			(9.00, 12.20)			(15.23, 16.51)	P < 0.001
West Area													
Maoming	1292	240	18.58	47	5	10.64	75	10	13.33	1414	255	18.03	χ^2^ = 3.1,
Huazhou			(16.47, 20.69)			(1.87, 19.41)			(5.68, 20.99)			(16.04, 20.03)	P > 0.05
Yangjiang	1393	355	25.48	74	4	5.41	54	8	14.81	1521	367	24.13	χ^2^ = 18.13,
Yangchun			(23.21, 27.76)			(0.28, 10.53)			(5.39, 24.24)			(21.99, 26.27)	P < 0.001
Zhanjiang	982	166	16.90	265	39	14.72	101	22	21.78	1348	227	16.84	χ^2^ = 2.62,
Lianjiang			(14.57, 19.24)			(10.47, 18.96)			(13.77, 29.79)			(14.85, 18.83)	P > 0.05
Total	3667	761	20.75	386	48	12.44	230	40	17.39	4283	849	19.82	χ^2^ = 16.11,
			(19.45, 22.06)			(9.16, 15.71)			(12.52, 22.26)			(18.63, 21.01)	P < 0.001
All Guangdong	20349	3491	17.16	5647	906	16.04	2483	289	11.64	28479	4686	16.45	χ^2^ = 49.85,
			(16.64, 17.67)			(15.09, 17.00)			(10.38, 12.89)			(16.03, 16.88)	P < 0.001

Note: CI is Confidence Interval.

### Detection of α- and β-globin gene mutations

Genomic DNA was extracted from all blood samples using a Lab-Aid 820 automation system (Zee San Biotech Company, Xiamen, Fujian, China). Twenty-three mutations of the a- and β-globin genes were identified using a Luminex 200 liquichip array system (R & D Systems Inc., Minneapolis, MN, USA) [6, 7]. The system was used throughout the process of probe design, the multiplex PCR, the attachment of probes to microspheres, and hybridization and analysis. The 23 thalassemia mutations included three α-globin gene deletions (i.e., the Southeast Asian deletion [-SEA], the rightward deletion [-α3.7], and the leftward deletion [-α4.2]). Three α-globin gene point mutations were also analyzed (i.e., Hb Constant Spring, Hb Quong Sze, and Hb Westmead). The 17 β-globin gene point mutations were on codons 41/42 (-TCTT), 654, -29 (A > G), -28 (A > G), codons 71/72 (+A), codon 17 (A > T), codon 43 (G > T), Hb E (β26[B8] Glu → Lys, GAG > AAG or codon 26 [G > A]), codons 27/28 (+C), codon 31 (-C), -32 (C > A), -30 (T > C), codons 14/15 (+G), IVS-I-1 (G > T), IVS-I-5 (G > T), Int, and Cap.

### Statistical analysis

We used the “vcd” and “car” program packages of R Version 3.0.1 statistical software (May 16, 2013, copyright 2013, R Foundation for Statistical Computing, Vienna, Austria) to analyze the data. We calculated the values for thalassemia carrier prevalence, with 95% confidence intervals (95% CIs), for the different household registry-based populations for all of the cities in Guangdong. The differences between prevalence values were compared using the chi-square test or the Mantel–Haenszel chi-square test. A thalassemia carrier was defined as an individual who carried any one of the 23 types of α- and/or β-globin gene mutations.

## Results

Blood samples were collected from a total of 28,479 individuals; 71.45% (20,349/28,479) were native residents of the city in which they resided, 19.83% (5,647/28,479) of them were migrants from another city within the province, and 8.72% (2,483/28,479) of them were immigrants from other provinces.

The results for the prevalence values for the different thalassemia genotypes are presented in Table 2. The overall prevalence of thalassemia carriers was 16.45%. The prevalence of α-, β-, and combined α-/β- thalassemia carriers was 12.03%, 3.80%, and 0.63%, respectively. The specific prevalence values for the different clinical forms of the disease were also calculated. The prevalence of α^+^-thalassemia was 5.71%, α^0^-thalassemia was 6.12%, hemoglobin H (HbH) disease was 0.20%, β-thalassemia minor (β^0^/β^N^ or β^+^/β^N^) was 3.78%, β-thalassemia intermedia was 0.01%, α^0^- or α^+^-thalassemia with β-thalassemia minor was 0.60%, HbH disease with β-thalassemia minor was 0.02%, α^0^-thalassemia with β-thalassemia intermedia carrier was 0.004%. The top three genotypes of α-thalassemia were --^SEA^/αα, α^3.7^α/αα, and α^4.2^α/αα. The top three genotypes of β-thalassemia were β^41–42^/β^N^, β^654^/β^N^, and β^-28^/β^N^.

**Table 2:** **The prevalence (%) of different genotypes of thalassemia among pregnant coupes of Guangdong, China (n = 28479)**

Individuals only with α-thalassemia			Individuals only with β-thalassemia			Individuals combined α-/β- thalassemia carriers					
Genotypes	Frequency	Prevalence (%95 CI)	Genotypes (clinic forms)	Frequency	Prevalence (%95 CI)	Genotypes (clinic forms)	Frequency	Prevalence (%95 CI)	Genotypes (clinic forms)	Frequency	Prevalence (%95 CI)
α^+^-thalassemia	1626	5.71% (5.44, 5.98)	β-thalassemia minor	1077	3.78 (3.56, 4.00)	--^SEA^/αα, β^41–42^/β^N^ (α^0^, β^0^/β^N^)	34	0.12 (0.08,0.16)	--^SEA^/α^3.7^, β^41–42^/β^N^ (HbH, β^0^/β^N^)	1	0.00 (0.00,0.01)
α^3.7^α/αα	911	3.20 (3.00, 3.40)	β^41–42^/β^N^ (β^0^/β^N^)	415	1.46 (1.32, 1.60)	--^SEA^/αα, β^654^/β^N^ (α^0^, β^0^/β^N^)	25	0.09 (0.05,0.12)	--^SEA^/αα, β^-28^/β^IVS-1^ (α^0^, β^+^/β^0^)	1	0.00 (0.00,0.01)
α^4.2^α/αα	313	1.10 (0.98, 1.22)	β^654^/β^N^ (β^0^/β^N^)	281	0.99 (0.87, 1.10)	α^3.7^/αα, β^41–42^/β^N^ (α^+^, β^0^/β^N^)	20	0.07 (0.04,0.10)	--^SEA^/αα, β^-29^/β^N^ (α^0^, β^+^/β^N^)	1	0.00 (0.00,0.01)
α^WS^α/αα	239	0.84 (0.73, 0.94)	β^17^/β^N^ (β^0^/β^N^)	95	0.33 (0.27, 0.40)	α^3.7^/αα, β^-28^/β^N^ (α^+^, β^+^/β^N^)	11	0.04 (0.02,0.06)	--^SEA^/αα, β^IVS-5^/β^N^ (α^0^, β^+^/β^N^)	1	0.00 (0.00,0.01)
α^CS^α/αα	83	0.29 (0.23, 0.35)	β^71–72^/β^N^ (β^0^/β^N^)	24	0.08 (0.05, 0.12)	--^SEA^/αα, β^-28^/β^N^ (α^0^, β^+^/β^N^)	9	0.03 (0.01,0.05)	α^3.7^/α^CS^α, β^41–42^/β^N^ (α^0^, β^0^/β^N^)	1	0.00 (0.00,0.01)
α^QS^α/αα	47	0.17 (0.12, 0.21)	β^27–28^/β^N^ (β^0^/β^N^)	19	0.07 (0.04, 0.10)	α^3.7^/αα, β^654^/β^N^ (α^+^, β^0^/β^N^)	8	0.03 (0.01,0.05)	α^3.7^/αα, β^-29^/β^N^ (α^+^, β^+^/β^N^)	1	0.00 (0.00,0.01)
α^3.7^α/α^4.2^α	9	0.03 (0.01, 0.05)	β^14–15^/β^N^ (β^0^/β^N^)	10	0.04 (0.01, 0.06)	α^WS^α/αα, β^41–42^/β^N^ (α^+^, β^0^/β^N^)	7	0.02 (0.01,0.04)	α^3.7^/αα, β^IVS-1^/β^N^ (α^+^, β^0^/β^N^)	1	0.00 (0.00,0.01)
α^3.7^α/α^3.7^α	8	0.03 (0.01, 0.05)	β^43^/β^N^ (β^0^/β^N^)	6	0.02 (0.00, 0.04)	α^4.2^/αα, β^41–42^/β^N^ (α^+^, β^0^/β^N^)	6	0.02 (0.00,0.04)	α^CS^α/αα, β^17^/β^N^ (α^+^, β^0^/β^N^)	2	0.01 (0.00,0.02)
α^3.7^α/α^WS^α	5	0.02 (0.00, 0.03)	β^IVS-1^/β^N^ (β^0^/β^N^)	6	0.02 (0.00, 0.04)	--^SEA^/αα, β^17^/β^N^ (α^0^, β^0^/β^N^)	4	0.01 (0.00,0.03)	α^CS^α/αα, β^654^/β^N^ (α^+^, β^0^/β^N^)	2	0.01 (0.00,0.02)
α^3.7^α/α^CS^α	3	0.01 (0.00, 0.02)	β^Int^/β^N^ (β^0^/β^N^)	1	0.00 (0.00, 0.01)	--^SEA^/αα, β^E^/β^N^ (α^0^, β^+^/β^N^)	4	0.01 (0.00,0.03)	α^3.7^/αα, β^IVS-5^/β^N^ (α^+^, β^+^/β^N^)	1	0.00 (0.00,0.01)
α^3.7^α/α^QS^α	3	0.01 (0.00, 0.02)	β^-28^/β^N^ (β^+^/β^N^)	155	0.54 (0.46, 0.63)	α^3.7^/αα, β^17^/β^N^ (α^+^, β^0^/β^N^)	4	0.01 (0.00,0.03)	α^4.2^/αα, β^71–72^/β^N^ (α^+^, β^0^/β^N^)	1	0.00 (0.00,0.01)
α^4.2^α/α^WS^α	3	0.01 (0.00, 0.02)	β^E^/ β^N^ (β^+^/β^N^)	24	0.08 (0.05, 0.12)	α^4.2^/αα, β^-28^/β^N^ (α^+^, β^+^/β^N^)	4	0.01 (0.00,0.03)	α^4.2^/αα, β^Cap^/β^N^ (α^+^, β^+^/β^N^)	1	0.00 (0.00,0.01)
α^4.2^α/α^CS^α	1	0.00 (0.00, 0.01)	β^Cap^/β^N^ (β^+^/β^N^)	23	0.08 (0.05, 0.11)	α^CS^α/αα, β^41–42^/β^N^ (α^+^, β^0^/β^N^)	4	0.01 (0.00,0.03)	α^CS^α/αα, β^-28^/β^N^ (α^+^, β^+^/β^N^)	1	0.00 (0.00,0.01)
α^4.2^α/α^QS^α	1	0.00 (0.00, 0.01)	β^-29^/β^N^ (β^+^/β^N^)	17	0.06 (0.03, 0.09)	α^3.7^/αα, β^71–72^/β^N^ (α^+^, β^0^/β^N^)	3	0.01 (0.00,0.02)	α^WS^α/αα, β^71–72^/β^N^ (α^+^, β^0^/β^N^)	1	0.00 (0.00,0.01)
α^0^-thalassemia	1743	6.12 (5.84, 6.40)	β^IVS-5^/β^N^ (β^+^/β^N^)	1	0.00 (0.00, 0.01)	α^4.2^/αα, β^17^/β^N^ (α^+^, β^0^/β^N^)	3	0.01 (0.00,0.02)	α^WS^α/αα, β^E^/β^N^ (α^+^, β^+^/β^N^)	1	0.00 (0.00,0.01)
--^SEA^/αα	1743	6.12 (5.84, 6.40)	β-thalassemia intermedia	4	0.01 (0.00, 0.03)	α^4.2^/αα, β654/β^N^ (α^+^, β^0^/β^N^)	3	0.01 (0.00,0.02)	α^WS^α/αα, β^IVS-1^/β^N^ (α^+^, β^0^/β^N^)	1	0.00 (0.00,0.01)
HbH disease	58	0.20 (0.15, 0.26)	β^-28^/β^E^ (β^+^/β^+^)	1	0.00 (0.00, 0.01)	--^SEA^/α^3.7^, β^654^/β^N^ (HbH, β^0^/β^N^)	2	0.01 (0.00,0.02)			
--^SEA^/α^3.7^	33	0.12 (0.08, 0.16)	β^41–42^/β^Cap^ (β^0^/β^+^)	1	0.00 (0.00, 0.01)	--^SEA^/α^CS^α, β^41–42^/β^N^ (HbH, β^0^/β^N^)	2	0.01 (0.00,0.02)			
--^SEA^/α^4.2^	15	0.05 (0.03, 0.08)	β^41–42^/β^E^ (β^0^/β^+^)	1	0.00 (0.00, 0.01)	--^SEA^/αα, β^71–72^/β^N^ (α^0^, β^0^/β^N^)	2	0.01 (0.00,0.02)			
--^SEA^/α^WS^α	5	0.02 (0.00, 0.03)	β^-28^/β^17^ (β^+^/β^0^)	1	0.00 (0.00, 0.01)	α^4.2^/αα, β^E^/β^N^ (α^+^, β^+^/β^N^)	2	0.01 (0.00,0.02)			
--^SEA^/α^CS^α	3	0.01 (0.00, 0.02)				α^WS^α/αα, β^654^/β^N^ (α^+^, β^0^/β^N^)	2	0.01 (0.00,0.02)			
--^SEA^/α^QS^α	2	0.01 (0.00, 0.02)				--^SEA^/α^3.7^, β^17^/β^N^ (HbH, β^0^/β^N^)	1	0.00 (0.00,0.01)			
Total	3427	12.03 (11.66, 12.41)	Total	1081	3.80 (3.57,4.02)	Total				178	0.63 (0.53,0.72)

Notes: 1. CI is Confidence Interval. 2. Hb H disease means Hemoglobin H (HbH) disease. 3. β^N^ means normal β-globin allele gene.

The results for thalassemia carrier prevalence in different population groups are presented in Table 1. The values for thalassemia carrier prevalence for the native city residents, the migrants from within the province, and the immigrants from outside the province were 17.16% (3491/20349), 16.04% (906/5647), and 11.64% (289/2483), respectively. Compared with the native city residents, the migrants from within the province and the immigrants from outside the province had a lower prevalence of thalassemia carriers, but the prevalence was >11%. The difference between these prevalence values was significant (χ^2^ = 49.85, *P* < 0.001).

For thalassemia carrier prevalence in the different areas of Guangdong Province, the differences between the different household registry populations were variable. Prevalence values were not significantly different (*P* > 0.05) between the four cities in the east area. In the mountain area, two of five cities had a significantly different prevalence (*P* < 0.05). In the Pearl River delta area, six of nine cities had a significantly different prevalence (*P* < 0.05), and in seven cities migrants from within the province had a higher prevalence of thalassemia carriers. In the west area, one of three cities had a significantly different prevalence value (*P* < 0.05).

## Discussion

Our results indicated that thalassemia is a serious public health problem in Guangdong. The average prevalence of thalassemia in the Guangdong population was 16.45%, and the prevalence of α-, β-, and combined α-/β- thalassemia was 12.03%, 3.80%, and 0.63%, respectively. These values were higher than values previously reported for prevalence for Guangdong Province [8, 9]. Our data also indicated that the native city resident population in the province, the migrant population from within the province, and the immigrant population from outside the province also have had a high prevalence of thalassemia. In some surveyed cities, the thalassemia prevalence in the migrant population from within the province was higher compared with the prevalence in native city residents in the province. In all targeted populations, the native city residents and the immigrants from other cities of the same or other provinces belong to the same ethnic group, and the finding that there were similar prevalences of thalassemia mutations supports this observation.

Worldwide, 56,000 conceptions per year have a major thalassemia disorder. Approximately 30,000 of these are affected by β-thalassemia major, and 3,500 succumb from hydrops fetalis syndrome during the perinatal period [1]. Interestingly, in Guangdong, thalassemia also causes a significant economic and disease burden. Thalassemias were originally characteristic of the tropics and subtropics, but are now common worldwide because of migration [10, 11]. Because thalassemia can be controlled in a cost-effective manner using programs that integrate treatment with carrier detection and genetic counseling, the World Health Organization has recommended global development of these services [12]. Many countries and areas have implemented antenatal or premarital thalassemia screening programs [13, 14]. Since the 1990s, some hospitals have delivered voluntary antenatal thalassemia screening and counseling services in Guangdong Province [14]. In 2011, the Guangdong government initiated an antenatal thalassemia prevention and control program, which includes 35 million RMB per year in financial support.

Because of the household registry arrangement and resource limitations, public health services for thalassemia prevention have only been offered to native city residents in the province. However, our results indicate that all segments of the Guangdong population should be screened for thalassemia. The migrants from within the province and the immigrants from outside the province accounted for nearly 30% of the total population in many cities in Guandong, which could affect the outcomes of thalassemia prevention programs and challenge the public health service system of Guangdong. The results of a Thalassemia International Federation study indicated that countries with a strong prevention and treatment program are well prepared to face the problem of hemoglobinopathy gene migration into the population [14].

Previously, the thalassemia prevalence reported for Guangdong Province was approximately 5–11% [9, 10]. The differences between these figures and our results were mainly owing to differences in areas selected for sampling, the populations examined, the detection methods, and the spectra of globin gene mutations examined. We sampled a greater number of cities and populations. We used the more sensitive liquichip array system for detection of globin gene mutation and found 23 types of globin gene mutations. A higher prevalence of thalassemia carriers (24.51%) has been reported for Guangxi Province [15]. Bangalore et al. reported that the prevalence of the β-thalassemia trait among pregnant women was 8.5%, [16] which was also higher than that of Guangdong, but these results were estimated from data from an antenatal care clinic.

The analysis of our data revealed that 0.22% (64/28479) of individuals were carriers of HbH disease, which could be a serious health problem for pregnant couples. The incidence of HbH disease has been reported to be 0.43% in Guangxi Province [15]. HbH disease is predominantly seen in South East Asia, the Middle East, and the Mediterranean [17].

There are some limitations that must be taken into consideration in our study. We only sampled one county or district in each city, so the results might not represent the true prevalence of thalassemia carriers in the population of that city. We also did not consider the duration of residence of the migrant populations.

## Conclusion

The prevalence of thalassemia gene mutations was high in native city resident populations in Guangdong Province, the migrant populations from within the province, and the immigrant populations from outside the province. The results indicate that all segments of the Guangdong population should be screened for thalassemia. The effectiveness of thalassemia prevention programs might be improved if the entire population in Guangdong Province were included. Policy makers should establish a strategy that allows affordable thalassemia prevention public health programs to be provided to inter-city and inter-province migrant populations in Guangdong Province.

### Ethical consideration

Ethical approval was obtained from Guangdong Women and Children Hospital Ethics Committee.
